# Using the Cardio-Ankle Vascular Index (CAVI) or the Mathematical Correction Form (CAVI_0_) in Clinical Practice

**DOI:** 10.3390/ijms21072410

**Published:** 2020-03-31

**Authors:** Javad Alizargar, Nan-Chen Hsieh, Shu-Fang Vivienne Wu, Shih-Yen Weng

**Affiliations:** 1 Research Center for Healthcare Industry Innovation, National Taipei University of Nursing and Health Sciences, Taipei City 112, Taiwan; 8shihyen@ntunhs.edu.tw; 2 Department of Information Management, National Taipei University of Nursing and Health Sciences, Taipei City 112, Taiwan; nchsieh@ntunhs.edu.tw; 3 College of Nursing, School of Nursing, National Taipei University of Nursing and Health Sciences, Taipei City 112, Taiwan; shufang@ntunhs.edu.tw

Tonhajzerova et al. [1] published a review paper in the *International Journal of Molecular Sciences* in which they evaluated atherosclerosis and other pathophysiological mechanisms involved in cardiovascular disease (CVD) in patients with human papillomavirus (HPV). The paper explored new biomarkers that could be used for affected individuals. They mentioned the cardio-ankle vascular index (CAVI), proposed by Shirai et al. [2] in 2006, as a method that can be used to assess the overall stiffness of arteries and provide information about the status of atherosclerosis in patients (Equation (1)). Although CAVI can be a proper stiffness index from the origin of the aorta to the ankle, there are some points that should be considered before using this index (and the indices associated with CAVI) in clinical practice.

CAVI_0_ (Equation (2)) is the mathematically corrected formula that has been derived from the CAVI formula. Spronck et al. [3] claimed that this marker is less dependent on blood pressure changes in the patient. In another article that was recently published, they also provided another tool to easily convert CAVI into CAVI_0_ values [4]. It is worth noting that CAVI_0_ has been doubted by the developer of CAVI because it has some flaws and cannot provide accurate measurements of arterial stiffness.

CAVI = a {(2ρ/∆P) × ln (Ps/Pd) PWV^2^} + b (1)

CAVI_0_=2ρ × (PWV^2^/P_d_) − ln (P_d_/P_0_) (2)

where PWV: pulse wave velocity of the arterial tree from the origin of the aorta to the ankle; P_s_: systolic blood pressure; Pd: diastolic blood pressure; ρ: blood density; Δ P: Ps – Pd; a, b: coefficients; P_0_: reference pressure (100 mmHg).

The use of CAVI_0_ was proposed by Spronck et al. [3] in 2017. They used data from 497 subjects to modify the original CAVI formula (by Shirai et al. [2]). There are some fundamental differences between CAVI and CAVI_0_. Unlike CAVI, which uses P_d_ and P_s_ in its formula, in calculating CAVI_0_, only P_d_, not P_s_, is used. Another difference between CAVI and CAVI_0_ is the use of P_0_ in the formula of CAVI_0_. Although Spronck et al. [3] claimed that the new formula of CAVI_0_ is independent of blood pressure, in a large study on 5293 individuals, Shirai et al. [5] recently showed that CAVI_0_ is not accurate because of its strong dependency on P_d_. Another reason that CAVI_0_ provides equivocal results is that Spronck et al. [3] did not take into account the physiological properties of individuals, such as their body mass index (BMI). BMI has been shown to be inversely associated with CAVI [6,7].

It is also interesting to know that the information about CAVI has been recently updated. Takahashi et al. [8] showed that the CAVI without the coefficients (coefficients a and b in Equation (1)) is also a valid index of arterial stiffness (Equation (3)). CAVI without coefficients a and b is parameter β, which is referred to as haβ (heart to ankle beta). Takahashi et al. [8] also revealed the coefficient “a” and “b” values that are being used in the CAVI calculation (Table 1). A review of this information raised some interesting discussion about the validity and use of the CAVI index. A study that aimed to simulate the influence of adjusting the coefficients in the equation of CAVI by Ato D. [9] suggested that the developers of CAVI fix these coefficients or terminate the use of CAVI. The reason is that those coefficients are dependent on the level of haβ (Table 1), and CAVI underestimates the original value of parameter β (haβ) that is used in the CAVI formula. Because the CAVI concept is driven by haβ (Equation (4)), it might cause inaccuracy in the calculation of the CAVI values.

haβ = (2ρ/∆P) × ln (Ps/Pd) haPWV^2^ (3)

CAVI= a haβ + b(4)

where haPWV: heart-ankle PWV.

As there are many concerns about using CAVI and CAVI_0_, we can say that PWV and parameter β can be considered as more reliable indices. As Tonhajzerova et al. [1] mentioned, PWV can still be considered an important risk marker, but given the fact that PWV and parameter β only include the more elastic aortic arterial wall and do not include the more muscular arm and leg arterial beds, researchers consider the use of more wholistic indices such as haPWV or haβ instead of PWV and parameter β, especially in older and high-risk patients, as muscular arteries are more likely to be affected by atherosclerosis after the proximal elastic arteries are affected [10]. The use of PWV and parameter β might yield similar results to haPWV and haβ in younger individuals.

Blood pressure is dependent on arterial stiffness (as a result of both structural and functional mechanisms) and arterial stiffness can be accelerated in the presence of hypertension [11]. Considering these facts, it is important to know which factors influence CAVI as an index of arterial stiffness. A recent study by Kamon et al. [11] showed that the blood pressure (BP) category was only associated with high CAVI in males, not females. This further emphasizes the role of sex along with age, diabetes and obesity in the management of hypertension.

## Figures and Tables

**Table 1:** The values of coefficients a and b in the cardio-ankle vascular index (CAVI) formula [8].

	haβ < 7.34875	7.34875 ≤ haβ < 10.30372	10.30372 ≤ haβ
Coefficient a	0.85	0.658	0.432
Coefficient b	0.695	2.103	4.41

## Abbreviations

CVD	Cardiovascular Disease
HPV	Human Papillomavirus
CAVI	Cardio-Ankle Vascular Index
BMI	Body Mass Index
P_s_	Systolic Blood Pressure
P_d_	Diastolic Blood Pressure
PWV	Pulse Wave Velocity
haPWV	Heart-Ankle PWV
haβ	Heart-Ankle Parameter β
